# Thymus and *Leishmania* at the Crossroads: Autoimmunity and Cancer

**DOI:** 10.1111/imm.70150

**Published:** 2026-05-15

**Authors:** Alef Batista Bezerra Barros, Gabriel Augusto Leite, Arthur Gomes de Andrade, Maria Danielma dos Santos Reis, Luiz Henrique Agra Cavalcante‐Silva, Marvin Paulo Lins

**Affiliations:** ^1^ Laboratory of Cell Biology Institute of Biological and Health Sciences, Federal University of Alagoas Maceió Brazil; ^2^ Medical School University of Cuiabá Mato Grosso Brazil; ^3^ Biotechnology Center Federal University of Paraíba João Pessoa Brazil; ^4^ Brazilian National Institute of Science and Technology on Neuroimmunomodulation (INCT‐NIM), Oswaldo Cruz Institute, Oswaldo Cruz Foundation Rio de Janeiro Brazil; ^5^ Medical Sciences and Nursing Complex Federal University of Alagoas Arapiraca Brazil; ^6^ Laboratory of Immunology, Department of Basic Sciences in Health Medical School, Federal University of Mato Grosso Cuiabá Brazil

**Keywords:** autoimmunity, cancer susceptibility, immune tolerance, *Leishmania* infection, thymic dysfunction

## Abstract

*Leishmania* infections profoundly reshape host immunity, yet their impact on central T‐cell development remains underappreciated. The thymus, a key site for establishing a functional and self‐tolerant T‐cell repertoire, is highly sensitive to systemic inflammatory cues. Growing evidence indicates that parasitic diseases, including leishmaniasis, can perturb thymic structure and function through direct parasite presence or sustained inflammatory stress. Such disturbances may alter thymocyte maturation, leading to reduced T‐cell output and the release of cells with aberrant specificity. Beyond compromising protective immunity, these changes may create conditions favourable to immune dysregulation, including the emergence of autoimmune manifestations and a microenvironment permissive to malignant transformation. This narrative review synthesises current findings on how *Leishmania* interacts with immunological tissues and highlights the thymus as a potential and overlooked target whose dysfunction may bridge infection, autoimmunity and cancer.

## Graphical abstract

Conceptual ‘Crossroads Model’ linking *Leishmania*, thymic infection and immune dysregulation: an integrative model is proposed in which *Leishmania* infection disrupts thymic architecture and stromal–thymocyte interactions. These alterations converge to promote defective tolerance, increased autoimmune risk and a microenvironment permissive to malignant transformation, driven by insufficient immunovigilance.

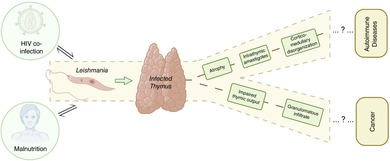

## 1 Introduction

The thymus is a lymphoid organ that plays a central role in the immune system, being responsible for the maturation and generation of functional and self‐tolerant T cells. This process relies on strict mechanisms of positive and negative selection, in which thymocytes capable of adequately recognising self‐MHC molecules are preserved. In contrast, those with high affinity for self‐antigens are eliminated to ensure self‐tolerance [1]. Thymic involution is a process frequently triggered during systemic infections. It is characterised by the progressive loss of immature T lymphocytes and stromal cells that are essential for the organ's architecture. This disruption leads to a reduction in thymic size and weight, accompanied by a marked decline in thymopoiesis, and consequently, a diminished output of competent T cells, ultimately compromising peripheral immune responses [2].

Within this broader context, *Leishmania* infection is particularly noteworthy. The parasite exhibits a pronounced tropism for immunological tissues, contributing to systemic alterations that include the thymic atrophy described in other infections. Growing evidence suggests that this tropism for immunoregulatory tissues may also extend to the thymus, fostering mechanisms of thymic dysfunction and contributing to imbalances in the T‐cell repertoire [3, 4]. These observations suggest that the influence of *Leishmania* on the thymus extends far beyond transient immune impairment. The presence of the parasite, or the systemic inflammatory milieu it induces, may compromise fundamental processes of thymic selection, favouring the escape of autoreactive and dysfunctional T lymphocytes and thereby increasing susceptibility to autoimmunity and the development of neoplasms. For these reasons, this review investigates the interaction between *Leishmania* and the thymus as a critical conceptual intersection, providing an integrated analysis of the interconnected processes involved.

This study is a narrative review. A comprehensive literature search was conducted in PubMed using combinations of keywords related to ‘*Leishmania*’, ‘thymus’, ‘autoimmunity’ and ‘cancer’. Articles were selected based on their relevance to the proposed theme, prioritising experimental and clinical studies addressing thymic alterations, immune modulation and disease outcomes. No formal systematic review protocol (e.g., PRISMA) was applied; however, efforts were made to ensure broad coverage of the available literature and inclusion of seminal and recent studies.

## 2 Thymus: Biology, Function and Plasticity

The thymus is a primary lymphoid organ located in the anterior mediastinum, composed of two asymmetric lobes enclosed by a fibrous capsule that subdivides into smaller lobules. Its architecture is organised into two main histological regions: the outer cortex and the inner medulla. The organ's morphological features vary according to cellular density, reflecting the maturation status of thymic populations. The thymic stroma is primarily composed of a specialised network of thymic epithelial cells (TECs), classically classified into cortical (cTECs) and medullary (mTECs), but also encompassing additional TEC subsets described in recent studies. In addition, the thymic microenvironment includes other stromal and haematopoietic cells, such as macrophages, dendritic cells and fibroblasts [5, 6].

During the generation of immunocompetent T cells, TECs play an essential role in guiding thymocyte maturation. Within the thymic microenvironment, thymocytes undergo sequential stages of differentiation and gene rearrangement to assemble a functional T cell receptor (TCR) [7], while TECs orchestrate key processes such as self‐MHC restriction (positive selection) and the deletion of autoreactive clones (negative selection) [8]. Thymocytes initially lack CD4 and CD8 expression and are thus classified as double‐negative; they subsequently become double‐positive following expression of the TCR β‐chain. Ultimately, fully matured thymocytes commit to either the CD4^+^ or CD8^+^ lineage [9].

mTECs are uniquely equipped to express tissue‐restricted antigens through the coordinated action of the transcription factors AIRE and FEZF2, which together broaden the spectrum of self‐representation and enable the efficient deletion of autoreactive clones as well as the diversion of some thymocytes into the regulatory T cell (Treg) lineage. AIRE promotes the ectopic expression of peripheral antigens and facilitates high‐avidity negative selection, whereas FEZF2 drives a complementary and partially nonoverlapping gene program essential for a complete self‐antigen landscape [10]. Thymic dendritic cells amplify this tolerogenic process by acquiring antigens from mTECs and presenting them through distinct antigen‐processing pathways, thereby enhancing both clonal deletion and Treg induction [11].

Impairments in thymic function, including defects in positive and negative selection, reduced diversification of the T cell repertoire or inadequate induction of thymic Tregs, undermine central tolerance and promote the escape of autoreactive clones [12]. Moreover, disturbances in thymic selection may diminish the generation of T cells with optimal TCR diversity and functional competence, thereby impairing immunovigilance against transformed cells. A narrowed or dysregulated T cell repertoire limits the immune system's ability to recognise neoantigens and mount effective antitumor responses, ultimately increasing susceptibility to malignancies [13]. Also, adult thymectomy might increase the long‐term risk of cancers or autoimmune diseases, impacting overall mortality [14].

Stress can disrupt thymic homeostasis, and several stress‐related conditions induce acute thymic involution, including emotional distress, malnutrition and pregnancy. Moreover, numerous pathological processes can trigger thymic atrophy, such as infections, inflammation, cancer therapy and conditioning regimens for bone marrow transplantation [15, 16]. A hallmark of parasitic diseases, including those caused by *Leishmania*, is the marked depletion of T cells [17]. In this sense, given that the thymus is essential for generating immunocompetent T cells capable of maintaining self‐tolerance while effectively eliminating neoplastic cells, dysfunctions in this organ are strongly linked to the development of autoimmunity and cancer. This highlights an important implication: effective treatment and eradication of primary parasitic infections may serve as a preventive strategy against future immunological complications.

## 3 Leishmania: Autoimmunity and Cancer

Leishmaniasis is a parasitic disease caused by protozoa of the genus *Leishmania*. The parasite is transmitted to humans through the bite of infected female sandflies of the *Phlebotomus* and *Lutzomyia* genera. Owing to its broad clinical spectrum and substantial socioeconomic impact, this disease represents a major public health challenge, particularly in tropical and subtropical regions. It affects an estimated 12 million people worldwide and is endemic in more than 90 countries. Visceral leishmaniasis is especially prevalent in Brazil, Ethiopia, India, Kenya, Somalia and Sudan, whereas cutaneous and mucocutaneous forms are widespread in Afghanistan, Pakistan, Syria, Saudi Arabia, Algeria, Iran, Brazil and Peru [18, 19].

Although the immune response is essential for controlling infections, infection‐driven immunity has been linked to the development of autoimmune phenomena [20]. Leishmaniasis, particularly its visceral forms, has been linked to both clinical and laboratory features of autoimmunity. Several studies have reported the production of autoantibodies, including anti‐DNA, anti‐Smith (anti‐Sm) and antiribonucleoprotein (RNP), in patients infected with *Leishmania* [21, 22, 23]. Supporting these findings, an *in silico* analysis by Múnera et al. [24] identified 33 human autoantigens associated with systemic lupus erythematosus (SLE) with at least 30% similarity to *Leishmania* sp. antigens. Indeed, multiple authors have described cases of visceral leishmaniasis mimicking the clinical and laboratory features of SLE [25, 26, 27]. Additional reports have also linked molecular mimicry in leishmaniasis to mixed cryoglobulinemia [28, 29].

In the pathogenesis of leishmaniasis, parasite‐induced tissue destruction is a defining feature. This process can release self‐antigens and promote epitope spreading, potentially triggering autoreactive T cell responses [30]. In addition, *Leishmania* can drive polyclonal B‐cell activation, leading to broad antibody production and hypergammaglobulinemia, which may further contribute to *Leishmania*‐associated autoimmunity [31]. Moreover, patients exhibit an increased frequency of CD4^+^CD25^high^FOXP3^+^ T cells in peripheral blood compared with healthy controls, and these cells upregulate IL‐10 production upon stimulation with *Leishmania* antigen [32]. Although this cytokine limits excessive inflammation, it is also implicated in driving the formation of pro‐inflammatory autoantibodies in SLE [33]. Thus, dysregulated IL‐10 responses may represent an additional mechanism linking leishmaniasis to autoimmune manifestations.

*Leishmania* infections have been associated with cancer through a range of direct and indirect host alterations; however, current evidence remains scarce and largely circumstantial (Table 1). Leishmaniasis can clinically mimic malignant conditions, often resulting in misdiagnosis or delays in appropriate management [43]. The cutaneous form may resemble malignancies such as Kaposi's sarcoma or cutaneous lymphoma [40, 44], whereas the visceral form can be confused with haematological cancers, including myelodysplastic syndrome and lymphoma [45, 46]. Chronic inflammation associated with *Leishmania* infection has been proposed as a potential contributor to carcinogenesis, although direct evidence remains lacking. Isolated observations, including basal cell carcinoma [38] and squamous cell carcinoma [36] arising in leishmanial scars, as well as hepatocellular carcinoma reported in patients with visceral disease [47], further suggest a possible association, but do not establish causality. Accordingly, this relationship requires more robust investigation. More recently, Larrea et al. [41] demonstrated that the BRCT domain of LmjPES, a *Leishmania major* homologue of the oncogene PES1, enhances tumour growth in mice and confers resistance to 5‐fluorouracil in HEK293T cells by modulating genes involved in proliferation, survival and chemoresistance. While preliminary, these findings provide initial experimental support for a potential interaction between leishmaniasis and cancer.

**TABLE 1:** Overview of studies reporting associations between *Leishmania* infection and cancer‐related outcomes.

References	Study type	*Leishmania* species	Model/clinical context	Cancer type/outcome	Nature of evidence	Key findings
[34]	Case report	Visceral leishmaniasis	Human (lymph node, bone marrow)	Burkitt lymphoma	Anecdotal/clinical observation	Coexistence of Burkitt lymphoma and visceral leishmaniasis in lymph node and bone marrow
[35]	Case report	*Leishmania donovani*	Human (bone marrow)	T‐cell prolymphocytic leukaemia	Anecdotal/clinical observation	Coexistence of leukaemia and *L. donovani* infection in bone marrow
[36]	Case report	*Leishmania major* (cutaneous)	Human (skin lesion)	Squamous cell carcinoma	Anecdotal/clinical observation	Squamous cell carcinoma arising in a chronic leishmanial scar
[37]	Case report	*Leishmania mexicana*	Human (skin lesion)	Epidermoid carcinoma	Anecdotal/clinical observation	Concurrent infection and carcinoma in the same tissue
[38]	Case report	Cutaneous leishmaniasis	Human (skin lesion)	Basal cell carcinoma	Anecdotal/clinical observation	Basal cell carcinoma developing in a long‐standing leishmanial scar
[39]	Case report	*L. donovani, Leishmania infantum*	Human (systemic infection)	T‐cell lymphoma/EBV‐associated lymphoproliferative disorder	Anecdotal/clinical observation	Immune dysregulation associated with leishmaniasis and EBV reactivation
[40]	Case report	*L. infantum*	Human (immunocompromised)	Pseudotumoral lesions	Mimicry/diagnostic overlap	Spindle‐cell pseudotumors mimicking sarcoma
[41]	In vitro/in vivo	*L. major*	HEK293T cells; mouse model	Malignant phenotype/drug resistance	Experimental (mechanistic)	PES1 homologue promotes tumour growth and chemoresistance
[42]	In vitro	*L. donovani*	Macrophages	Epigenetic alterations associated with cancer pathways	Indirect/mechanistic	Infection induces DNA methylation changes linked to oncogenic pathways

*Note*: This table summarises selected studies exploring potential associations between *Leishmania* infection and cancer‐related outcomes. The evidence presented is heterogeneous and includes case reports, observational findings and experimental studies. Importantly, most reports describe co‐occurrence or indirect mechanisms, and do not establish causality.

Additionally, evidence demonstrates that *Leishmania* infections (particularly those caused by *Leishmania donovani, Leishmania infantum and Leishmania braziliensis*) can induce aberrant DNA methylation patterns in macrophages and in cutaneous lesions [42, 48]. These epigenetic modifications have been proposed to increase susceptibility to malignant transformation by silencing tumour‐suppressor genes and activating oncogenic pathways, thereby promoting a microenvironment permissive to oncogenesis; however, their direct contribution in the context of leishmaniasis remains to be demonstrated. The long‐term persistence of the parasite within host tissues may sustain these alterations, potentially conferring prolonged cancer risk [49]. In parallel, the predominance of a Th2‐type immune response in cases of progressive leishmaniasis has been suggested to create a microenvironment that could favour tumour cell proliferation, although this remains speculative [50]. Collectively, these observations highlight the need for further investigation into the oncogenic potential of *Leishmania* and its possible relationship with thymic alterations.

## 4 Thymus Versus *Leishmania*

Few studies have examined the impact of *Leishmania* infection on the thymus in either animal models (*n* = 14) or humans (*n* = 2), as summarised in Table 1. The first scientific report was published in 1973 by Schnur et al., who identified amastigotes of *Leishmania tropica* and *L. donovani* within the thymus of Syrian hamsters ( *Mesocricetus auratus* ). In humans, the first description appeared in 1992, when Eltoum et al. reported *Leishmania* sp. in the thymus of a child during autopsy, attributed to congenital transmission from an infected mother. Since then, most studies have used BALB/c mice to investigate thymic alterations induced by *Leishmania* infection.

The available evidence indicates that the thymus is a significant target of *Leishmania* infection, resulting in morphofunctional damage that can compromise T‐cell development and, consequently, has implications for autoimmunity, susceptibility to secondary infections, and even oncogenesis. Overall, the presence of intrathymic amastigote emerges as a recurrent finding, with parasites observed free in the medulla and the corticomedullary junction [3], within macrophages [51], and even suggesting infection of TECs [52]. The infection also induces corticomedullary disorganisation, thymic involution, fibrosis, increased collagen deposition, hyperplasia, granulomatous inflammation and alterations in the expression of structural proteins such as vimentin and fibronectin (for references, see Table 2).

**TABLE 2:** Summary of thymic alterations reported in experimental and clinical studies of *Leishmania* infection.

Year	*Leishmania* species	Model (animal/human)	Outcomes in thymus	Notes	References
2023	*Leishmania infantum*	Syrian hamsters ( *Mesocricetus auratus* )	Amastigotes found free in the medulla and at the corticomedullary junction; thymic atrophy; increased vimentin deposition.		Março et al. [3]
2022	*L. infantum*	Dogs	Intrathymic amastigotes; thymic atrophy; increased vascularisation; loss of the corticomedullary junction; increased collagen deposition; reduced Ki67 expression in thymuses from infected animals.		Jussiani et al. [53]
2021	*Leishmani amazonensis*	Balb/C mice	Thymic hypertrophy and hyperplasia (increased cellular density); loss of corticomedullary demarcation; cortical reduction.		Arrais‐Lima et al. [54]
2021	*Leishmania donovani*	Balb/C mice	Intracellular amastigotes in the thymic medulla (appearing to localise within TEC cytoplasm); systemic parasite burden directly proportional to thymic infection; miltefosine treatment reduces thymic parasitism.	Detection of infection by imaging, highly noteworthy.	Domínguez‐Asenjo et al. [52]
2020	Visceral leishmaniasis	Human	Impaired thymic output associated with poor immune reconstitution and disturbances in the T‐cell repertoire in relapsing visceral leishmaniasis in HIV/AIDS patients.	HIV and *Leishmania* co‐infection.	Silva‐Freitas et al. [55]
2020	*L. infantum*	Dogs	Amastigotes inside macrophages in the thymic cortex and medulla; thymic involution; thymitis with granulomatous or pyogranulomatous inflammatory infiltrates; positive correlation between fibronectin deposition and clinical severity.		da Silva et al. [56]
2019	*L. infantum*	Balb/C mice	Reduction of total and double‐positive thymocytes; decreased Ki‐67 in DP cells; 17% of thymic interstitial fluid proteins downregulated and 3% upregulated.	Infected and malnourished.	Losada‐Barragán et al. [57]
2017	*L. infantum*	Balb/C mice	Reduction in total thymocytes and DP cells; increased thymic weight; amastigotes inside or outside macrophages in the thymic medulla; increased apoptosis of DP and CD4 thymocytes; increased CXCR3^+^CD8^+^ cells, intrathymic CXCL9 and CXCL10, and intrathymic CXCL12 and CCL5.	Infected and malnourished.	Losada‐Barragán et al. [58]
2016	*L amazonensis e L brasiliensis*	Balb/C mice	No changes in relative thymic weight or thymocyte subset distribution in infected animals.	Co‐infected with *Plasmodium*.	Pinna et al. [59]
2014	*L. infantum*	Balb/C mice	Relative thymic weight increased; absolute cellularity increased; percentage of DP thymocytes reduced; IL‐12a mRNA increased in thymocytes.	Infected and malnourished.	Cuervo Escobar et al. [17]
1994	*L. donovani*	Golden hamster ( *M. auratus* )	Decrease in reduced and oxidised glutathione levels.		Goyal et al. [60]
1994	*Leishmania major*	Balb/C mice	Based on tissue culture in blood agar plates, the thymus was not colonised by *Leishmania major*, and no increase in γδ T cells was observed in thymuses of infected mice.		Rosat et al. [61]
1993	*L. donovani e L. infantum*	African green monkeys (*Cercopithecus aethiops*)	Presence of macrophage clusters in the thymus, often containing amastigotes.		Binhazim et al. [51]
1992	*L. (L.) chagasi*	Hamsters ( *M. auratus* )	Thymus showing only mild cortical atrophy and some hyalinised Hassall bodies.		Corbett et al. [62]
1992	Visceral leishmaniasis	Human	*Leishmania* identified in the thymus of a child at autopsy, compatible with congenital transmission from an infected mother.		Eltoun et al. [63]
1973	*Leishmania tropica e L donovani*	Syrian hamsters ( *M. auratus* )	Amastigotes detected in the thymus.		Schnur et al. [64]

*Note*: This table compiles published findings describing structural, cellular and molecular changes observed in the thymus during *Leishmania* infection, across diverse species and experimental or clinical settings.

In models of concomitant malnutrition [17, 58], profound alterations in the thymic profile of proteins and reductions in thymocyte numbers (double‐negative compartment) have been observed, indicating a direct impact on T‐cell ontogeny. More recent studies add important nuances: Domínguez‐Asenjo et al. [52] demonstrated that systemic parasite burden correlates with thymic infection and that treatment with miltefosine reduces thymic parasitism; Pinna et al. [59] showed that *Plasmodium* coinfection can modify the classical pattern of thymic alterations; and studies in dogs [56] further highlight that inflammatory infiltration and extracellular matrix remodelling are directly associated with clinical severity. Additionally, evidence from patients coinfected with HIV and *Leishmania* [55] reveals a marked reduction in thymic output, suggesting that the human thymus is also functionally compromised under these conditions.

**FIGURE 1:**
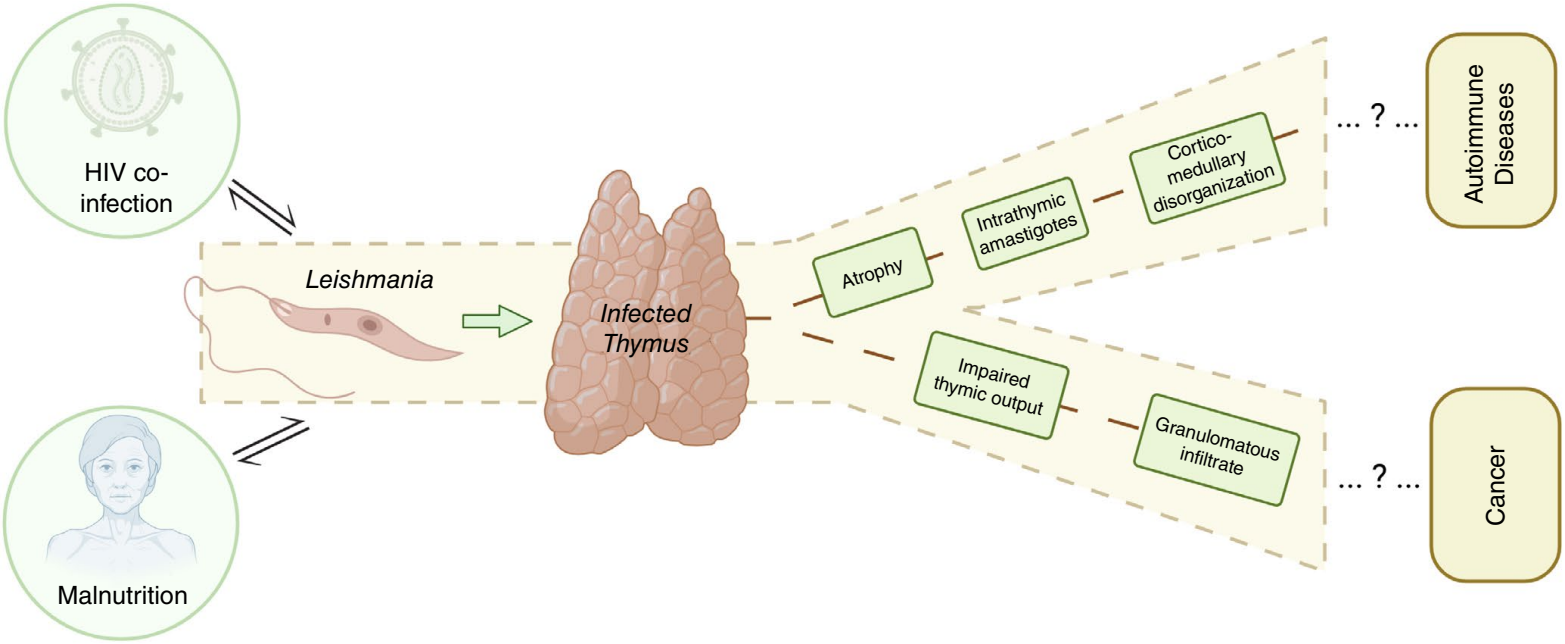
Conceptual ‘crossroads model’ linking *Leishmania*, thymic infection and immune dysregulation. In this scheme, an integrative model is proposed in which *Leishmania* infection disrupts thymic architecture and stromal–thymocyte interactions, impairing negative selection, thymocyte differentiation and T‐cell repertoire formation. These alterations converge to promote defective tolerance, increased autoimmune risk, and a microenvironment permissive to malignant transformation, driven by insufficient immunovigilance. (Created with BioRender.com).

Taken together, these data support an integrative framework in which *Leishmania* infection acts directly on the thymus, inducing tissue disorganisation, thymocyte depletion, altered chemokine expression and persistent intrathymic parasitism. Such defects facilitate the escape of autoreactive clones, contributing to the autoimmune manifestations documented in leishmaniasis, and also weaken antitumor immunosurveillance, creating conditions permissive to malignant transformation. Considered as a whole, these observations support the proposition of a ‘Crossroads Model’, in which thymic disruption represents a pivotal intersection linking *Leishmania*‐induced immunological imbalance to downstream risks of autoimmunity and cancer (Figure 1).

## 5 Perspectives of Therapy and Translation

Thymic function is compromised during *Leishmania* infections; therefore, structural and functional assessment tools could be employed to detect thymic atrophy and to monitor its recovery during and after infection. Although accurately evaluating thymic function in humans remains challenging, a combined approach integrating structural imaging modalities, flow‐cytometric analysis of T‐cell subset distribution, and quantification of T‐cell receptor excision circles (TRECs) provides a multidimensional framework to identify, track and better understand thymic impairment throughout the course of leishmaniasis [65].

Since thymic atrophy and impaired T‐cell output may contribute to long‐term immune dysregulation in affected individuals, strategies that preserve or restore thymic architecture could mitigate downstream consequences such as heightened susceptibility to secondary infections, autoimmunity and potentially, though still hypothetically, cancer. Experimental approaches under investigation in other contexts of thymic injury, including cytokine‐based interventions such as IL‐7, IL‐22 and KGF [66], modulation of endogenous repair pathways involving FOXN1 or BMP4 [67] and transient inhibition of sex steroids to stimulate thymopoiesis [68], may offer valuable translational insights. Although these interventions have not yet been evaluated in the setting of leishmaniasis, adapting and testing them in this context could clarify whether thymic support enhances immune reconstitution and whether this could translate into improved clinical outcomes.

In parallel, advances in vaccine development against *Leishmania* raise important questions about how thymic impairment influences long‐term protective immunity. Because *Leishmania*‐induced thymic disruption can impair TCR diversity and T‐cell differentiation, clarifying how this injury reshapes the balance between effector and regulatory T‐cells may help guide the refinement of vaccine design and adjuvant strategies [69]. Furthermore, evaluating whether thymus‐protective interventions [70] enhance vaccine efficacy may suggest a potential translational avenue: the combination of antiparasitic treatment, thymic‐support strategies and vaccination to achieve durable immunity [71]. Integrating these dimensions could contribute to future improvements in the clinical management of leishmaniasis and broaden the scope of host‐directed therapies.

## 6 Future Directions

To advance the understanding of how *Leishmania* affects thymic biology, new directions and experimental approaches can be proposed for future investigations. Beyond the traditional assessment of thymic mass, total cellular content, thymocyte subset distribution and routine histopathological examination aimed at detecting microarchitectural disturbances, additional methodologies may be warranted. In particular, expanded immunohistochemical panels may be employed to characterise components of the thymic extracellular matrix, including distinct collagen isoforms and laminin, as well as their principal interacting partners, such as integrins.

To expand current knowledge, it is equally important to incorporate evaluations focused on the nonhaematopoietic stromal compartment, especially TECs and thymic DCs. Detailed investigations of their structural features, patterns of soluble factor release, extracellular matrix contributions and global transcriptional activity (via RNA‐sequencing) could provide a substantial refinement of current insights. Thymocyte behaviour can also be examined through functional assays performed in vitro, including cytokine secretion tests, interactions with stromal or matrix substrates and migration analyses. Modern 3D culture platforms, including Fetal Thymic Organ Cultures (FTOCs), bioengineered thymic organoids or decellularised thymic matrices, offer promising systems to recreate thymic environments experimentally. In parallel, delineating the emergence of conventional T cells and Tregs, as well as mapping the diversity of the TCR repertoire, can be achieved using humanised models or animals engineered with targeted genetic modifications.

These experimental avenues may provide a conceptual and methodological roadmap for clarifying how *Leishmania*‐driven thymic infection reshapes central tolerance, perturbs stromal–lymphoid crosstalk and influences systemic immune outcomes. By applying these methods specifically to *Leishmania* models (murine, canine or humanised), future studies will be better positioned to describe the mechanisms by which the parasite disrupts thymopoiesis, promotes aberrant T‐cell selection and potentially contributes to autoimmunity or, more speculatively, neoplastic transformations emerging from a chronically dysregulated thymus.

## 7 Conclusion

Recognising the thymus as a critical target of infection reframes our understanding of host–parasite interactions and raises essential questions about how central tolerance, T‐cell repertoire formation and long‐term immune recovery are shaped during and after disease. Although most research has focused on peripheral immune alterations, accumulating evidence shows that *Leishmania* has a profound impact on thymic structure and function, leading to impaired thymopoiesis, dysregulated T‐cell selection and long‐lasting consequences for immune competence. Integrating thymic evaluation into studies of leishmaniasis, whether through imaging, cellular analyses, molecular profiling or functional assessments, is essential for advancing both basic immunology and translational strategies in *Leishmania* infections.

## Author Contributions

**Alef Batista Bezerra Barros:** investigation, methodology, writing – original draft. **Gabriel Augusto Leite:** investigation, methodology, writing – original draft. **Arthur Gomes de Andrade:** investigation, methodology, writing – original draft. **Maria Danielma dos Santos Reis:** writing – review and editing. **Luiz Henrique Agra Cavalcante‐Silva:** writing – review and editing, supervision. **Marvin Paulo Lins:** conceptualisation, writing – review and editing, supervision.

## Funding

The authors acknowledge the financial support for open access publication provided by the CAPES‐Wiley agreement.

## Conflicts of Interest

The authors declare no conflicts of interest.

## Data Availability

The data that support the findings of this study are available from the corresponding author upon reasonable request.

## References

[1] L. Klein , B. Kyewski , P. M. Allen , and K. A. Hogquist , “Positive and Negative Selection of the T Cell Repertoire: What Thymocytes See (and Don't See),” Nature Reviews Immunology 14, no. 6 (2014): 377–391, 10.1038/nri3667. PMC4757912 24830344

[2] M. Luo , L. Xu , Z. Qian , and X. Sun , “Infection‐Associated Thymic Atrophy,” Frontiers in Immunology 12 (2021): 652538, 10.3389/fimmu.2021.652538. 34113341 PMC8186317

[3] K. S. Março , J. da Silva Borégio , G. G. Jussiani , et al., “Thymic Alterations Resulting From Experimental Visceral Leishmaniasis in a Syrian Hamster ( *Mesocricetus auratus* ),” Veterinary Immunology and Immunopathology 257 (2023): 110558, 10.1016/j.vetimm.2023.110558. 36758455

[4] W. Savino , J. Durães , C. Maldonado‐Galdeano , G. Perdigon , D. A. Mendes‐da‐Cruz , and P. Cuervo , “Thymus, Undernutrition, and Infection: Approaching Cellular and Molecular Interactions,” Frontiers in Nutrition 9 (2022): 948488, 10.3389/fnut.2022.948488. 36225882 PMC9549110

[5] G. E. Marcovecchio , I. Bortolomai , F. Ferrua , et al., “Thymic Epithelium Abnormalities in DiGeorge and Down Syndrome Patients Contribute to Dysregulation in T Cell Development,” Frontiers in Immunology 10 (2019): 447. 30949166 10.3389/fimmu.2019.00447 PMC6436073

[6] P. Rouse , T. Henderson , S. Venkateswaran , et al., “An Induced Thymic Epithelial Cell‐Based High Throughput Screen for Thymus Extracellular Matrix Mimetics,” European Journal of Immunology 53, no. 3 (2023): e2249934, 10.1002/eji.202249934. 36645212

[7] J. Gommeaux , C. Grégoire , P. Nguessan , et al., “Thymus‐Specific Serine Protease Regulates Positive Selection of a Subset of CD4+ Thymocytes,” European Journal of Immunology 39, no. 4 (2009): 956–964, 10.1002/eji.200839175. 19283781

[8] P. M. Rodrigues , L. G. Sousa , C. Perrod , et al., “LAMP2 Regulates Autophagy in the Thymic Epithelium and Thymic Stroma‐Dependent CD4 T Cell Development,” Autophagy 19, no. 2 (2023): 426–439, 10.1080/15548627.2022.2074105. 35535798 PMC9851248

[9] J. Malin , G. U. Martinez‐Ruiz , Y. Zhao , et al., “Expression of the Transcription Factor Klf6 by Thymic Epithelial Cells Is Required for Thymus Development,” Science Advances 9, no. 46 (2023): eadg8126, 10.1126/sciadv.adg8126. 37967174 PMC10651122

[10] Y. Qi , R. Zhang , Y. Lu , X. Zou , and W. Yang , “Aire and Fezf2, Two Regulators in Medullary Thymic Epithelial Cells, Control Autoimmune Diseases by Regulating TSAs: Partner or Complementer?,” Frontiers in Immunology 13 (2022): 948259. 36110862 10.3389/fimmu.2022.948259 PMC9468217

[11] M. Vobořil , S. Xuan , and K. A. Hogquist , “Thymic Dendritic Cells Revisited,” Immunological Reviews 336, no. 1 (2025): e70076, 10.1111/imr.70076. 41251667 PMC12626115

[12] B. D. Coder , H. Wang , L. Ruan , and D. M. Su , “Thymic Involution Perturbs Negative Selection Leading to Autoreactive T Cells That Induce Chronic Inflammation,” Journal of Immunology 194, no. 12 (2015): 5825–5837. 10.4049/jimmunol.1500082 PMC4458423 25957168

[13] W. Wang , R. Thomas , O. Sizova , and D. M. Su , “Thymic Function Associated With Cancer Development, Relapse, and Antitumor Immunity—A Mini‐Review,” Frontiers in Immunology 11 (2020): 773, 10.3389/fimmu.2020.00773. 32425946 PMC7203483

[14] K. A. Kooshesh , B. H. Foy , D. B. Sykes , K. Gustafsson , and D. T. Scadden , “Health Consequences of Thymus Removal in Adults,” New England Journal of Medicine 389, no. 5 (2023): 406–417, 10.1056/NEJMoa2302892. 37530823 PMC10557034

[15] W. Savino and M. Dardenne , “Nutritional Imbalances and Infections Affect the Thymus: Consequences on T‐Cell‐Mediated Immune Responses,” Proceedings of the Nutrition Society 69, no. 4 (2010): 636–643, 10.1017/S0029665110002545. 20860857

[16] A. R. Ansari and H. Liu , “Acute Thymic Involution and Mechanisms for Recovery,” Archivum Immunologiae et Therapiae Experimentalis (Warsz) 65, no. 5 (2017): 401–420, 10.1007/s00005-017-0462-x. 28331940

[17] S. Cuervo‐Escobar , M. Losada‐Barragán , A. Umaña‐Pérez , et al., “T‐Cell Populations and Cytokine Expression Are Impaired in Thymus and Spleen of Protein Malnourished BALB/c Mice Infected With *Leishmania infantum* ,” PLoS One 9, no. 12 (2014): e114584. 25535967 10.1371/journal.pone.0114584 PMC4275170

[18] S. Burza , S. L. Croft , and M. Boelaert , “Leishmaniasis,” Lancet 392, no. 10151 (2018): 951–970, 10.1016/S0140-6736(18)31204-2. 30126638

[19] H. Kato , “Epidemiology of Leishmaniasis: Risk Factors for Its Pathology and Infection,” Parasitology International 105 (2025): 102999, 10.1016/j.parint.2024.102999. 39592080

[20] D. Johnson and W. Jiang , “Infectious Diseases, Autoantibodies, and Autoimmunity,” Journal of Autoimmunity 137 (2023): 102962. 36470769 10.1016/j.jaut.2022.102962 PMC10235211

[21] S. Argov , C. L. Jaffe , M. Krupp , H. Slor , and Y. Shoenfeld , “Autoantibody Production by Patients Infected With Leishmania,” Clinical and Experimental Immunology 76, no. 2 (1989): 190–197. 2788044 PMC1541839

[22] S. Lakhal , M. Benabid , I. B. Sghaier , J. Bettaieb , A. Bouratbine , and Y. Galai , “The Sera From Adult Patients With Suggestive Signs of Autoimmune Diseases Present Antinuclear Autoantibodies That Cross‐React With *Leishmania infantum* Conserved Proteins: Crude Leishmania Histone and Soluble Leishmania Antigens [Corrected],” Immunologic Research 61, no. 1–2 (2015): 154–159, 10.1007/s12026-014-8589-x. 25395341

[23] E. Liberopoulos , A. Kei , F. Apostolou , and M. Elisaf , “Autoimmune Manifestations in Patients With Visceral Leishmaniasis,” Journal of Microbiology, Immunology, and Infection 46, no. 4 (2013): 302–305, 10.1016/j.jmii.2012.01.016. 22516744

[24] M. Múnera , J. Farak , M. Pérez , et al., “Prediction of Molecular Mimicry Between Antigens From Leishmania sp. and Human: Implications for Autoimmune Response in Systemic Lupus Erythematosus,” Microbial Pathogenesis 148 (2020): 104444, 10.1016/j.micpath.2020.104444. 32827635

[25] J. B. Arlet , L. Capron , and J. Pouchot , “Visceral Leishmaniasis Mimicking Systemic Lupus Erythematosus,” Journal of Clinical Rheumatology 16, no. 4 (2010): 203–204, 10.1097/RHU.0b013e3181dfd26f. 20511988

[26] G. C. L. Bueno , A. T. S. Koerich , L. B. Burg , S. L. Kretzer , J. Â. G. D. Moral , and I. A. Pereira , “Visceral Leishmaniasis Mimicking Systemic Lupus Erythematosus,” Revista da Sociedade Brasileira de Medicina Tropical 52 (2019): e20180208, 10.1590/0037-8682-0208-2018. 30810652

[27] L. I. Sakkas , M. Boulbou , D. Kyriakou , et al., “Immunological Features of Visceral Leishmaniasis May Mimic Systemic Lupus Erythematosus,” Clinical Biochemistry 41, no. 1–2 (2008): 65–68, 10.1016/j.clinbiochem.2007.10.008. 17991433

[28] M. Casato , F. G. de Rosa , L. P. Pucillo , et al., “Mixed Cryoglobulinemia Secondary to Visceral Leishmaniasis,” Arthritis and Rheumatism 42, no. 9 (1999): 2007–2011. 10513819 10.1002/1529-0131(199909)42:9<2007::AID-ANR30>3.0.CO;2-X

[29] E. Rizos , G. Dimos , E. N. Liberopoulos , M. S. Elisaf , and A. A. Drosos , “Cryoglobulinemic Purpura in Visceral Leishmaniasis,” Rheumatology International 25, no. 6 (2005): 469–471, 10.1007/s00296-004-0533-2. 16133583

[30] D. Liu and J. E. Uzonna , “The Early Interaction of Leishmania With Macrophages and Dendritic Cells and Its Influence on the Host Immune Response,” Frontiers in Cellular and Infection Microbiology 2, no. 2 (2012): 83, 10.3389/fcimb.2012.00083. 22919674 PMC3417671

[31] B. Galvão‐Castro , J. A. Sá Ferreira , K. F. Marzochi , M. C. Marzochi , S. G. Coutinho , and P. H. Lambert , “Polyclonal B Cell Activation, Circulating Immune Complexes and Autoimmunity in Human American Visceral Leishmaniasis,” Clinical and Experimental Immunology 56, no. 1 (1984): 58–66. 6424987 PMC1535977

[32] R. F. Peixoto , B. M. Gois , M. Martins , et al., “Characterization of Regulatory T Cells in Patients Infected by *Leishmania infantum* ,” Tropical Medicine and Infectious Disease 8, no. 1 (2022): 18, 10.3390/tropicalmed8010018. 36668925 PMC9864225

[33] F. Facciotti , P. Larghi , R. Bosotti , et al., “Evidence for a Pathogenic Role of Extrafollicular, IL‐10‐Producing CCR6+B Helper T Cells in Systemic Lupus Erythematosus,” Proceedings of the National Academy of Sciences of the United States of America 117, no. 13 (2020): 7305–7316. 32184325 10.1073/pnas.1917834117 PMC7132288

[34] N. Boutros , D. Hawkins , M. Nelson , I. A. Lampert , and K. N. Naresh , “Burkitt Lymphoma and Leishmaniasis in the Same Tissue Sample in an AIDS Patient,” Histopathology 48, no. 7 (2006): 880–881, 10.1111/j.1365-2559.2006.02434.x. 16722944

[35] H. Liao , Y. Jin , J. Yu , and N. Jiang , “Concomitant T‐Cell Prolymphocytic Leukemia and Visceral Leishmaniasis: A Case Report,” Medicine (Baltimore) 97, no. 38 (2018): e12410, 10.1097/MD.0000000000012410. 30235714 PMC6160114

[36] R. Friedman , S. Hanson , and L. H. Goldberg , “Squamous Cell Carcinoma Arising in a Leishmania Scar,” Dermatologic Surgery 29, no. 11 (2003): 1148–1149, 10.1046/j.1524-4725.2003.29354.x. 14641345

[37] S. D. Blum‐Domínguez , A. Martínez‐Vázquez , L. A. Núñez‐Oreza , F. Martínez‐Hernández , G. Villalobos , and P. Tamay‐Segovia , “Leishmaniasis cutánea Difusa (LCD) y Visceral (LV) Concurrentes Con cáncer: Presentación de Un Caso [Diffuse Cutaneous Leishmaniasis (DCL) and Visceral Leishmaniasis (VL) Concurrent With Cancer: Presentation of a Case],” Gaceta Médica de México 153, no. 1 (2017): 121–124. 28128815

[38] M. S. Gurel , L. Inal , I. Ozardali , and S. A. Duzgun , “Basal Cell Carcinoma in a Leishmanial Scar,” Clinical and Experimental Dermatology 30, no. 4 (2005): 444–445, 10.1111/j.1365-2230.2005.01780.x. 15953097

[39] N. M. Osakwe , A. Paulus , P. F. Haggerty , et al., “Visceral Leishmaniasis With Associated Immune Dysregulation Leading to Lymphoma,” Military Medicine 178, no. 3 (2013): e386–e389, 10.7205/MILMED-D-12-00407. 23707131

[40] C. Perrin , J. F. Michiels , E. Bernard , P. Hofman , E. Rosenthal , and R. Loubiere , “Cutaneous Spindle‐Cell Pseudotumors due to Mycobacterium Gordonae and *Leishmania infantum*. An Immunophenotypic Study,” American Journal of Dermatopathology 15, no. 6 (1993): 553–558, 10.1097/00000372-199312000-00007. 8311186

[41] E. Larrea , C. Fernández‐Rubio , J. Peña‐Guerrero , E. Guruceaga , and P. A. Nguewa , “The BRCT Domain From the Homologue of the Oncogene PES1 in *Leishmania major* (LmjPES) Promotes Malignancy and Drug Resistance in Mammalian Cells,” International Journal of Molecular Sciences 23, no. 21 (2022): 13203, 10.3390/ijms232113203. 36361992 PMC9655562

[42] A. K. Marr , J. L. MacIsaac , R. Jiang , A. M. Airo , M. S. Kobor , and W. R. McMaster , “ *Leishmania donovani* Infection Causes Distinct Epigenetic DNA Methylation Changes in Host Macrophages,” PLoS Pathogens 10, no. 10 (2014): e1004419. 25299267 10.1371/journal.ppat.1004419 PMC4192605

[43] P. Kopterides , E. G. Mourtzoukou , E. Skopelitis , N. Tsavaris , and M. E. Falagas , “Aspects of the Association Between Leishmaniasis and Malignant Disorders,” Transactions of the Royal Society of Tropical Medicine and Hygiene 101, no. 12 (2007): 1181–1189, 10.1016/j.trstmh.2007.08.003. 17870139

[44] S. Philibert‐Rosas , R. Rabago Escoto , A. H. Hernandez Lara , C. Tenorio Flores , and E. Ornelas Escobedo , “Gastrointestinal Kaposi: A Rare Case Unveiling the Presentation and Management Challenges of an Uncommon Neoplasm in the Digestive Tract,” Cureus 16, no. 3 (2024): e56892, 10.7759/cureus.56892. 38659566 PMC11042665

[45] M. Fakhar , Q. Asgari , M. H. Motazedian , and A. Monabati , “Mediterranean Visceral Leishmaniasis Associated With Acute Lymphoblastic Leukemia (ALL),” Parasitology Research 103, no. 2 (2008): 473–475, 10.1007/s00436-008-0999-z. 18463894

[46] A. Kawakami , T. Fukunaga , M. Usui , et al., “Visceral Leishmaniasis Misdiagnosed as Malignant Lymphoma,” Internal Medicine 35, no. 6 (1996): 502–506, 10.2169/internalmedicine.35.502. 8835605

[47] D. F. Precone , G. Stornaiuolo , D. Galante , A. Amato , L. Gradoni , and G. B. Gaeta , “Case Report: Effect of Antileishmanial Treatment on Hepatitis C Viraemia in a Visceral Leishmaniasis Patient With Chronic Hepatitis C,” Transactions of the Royal Society of Tropical Medicine and Hygiene 97, no. 5 (2003): 559–560, 10.1016/s0035-9203(03)80028-7. 15307426

[48] E. Loi , P. A. Barroso , A. Moya Alvarez , P. Zavattari , and A. F. Vega Benedetti , “DNA Methylation in Macrophages Infected With Leishmania spp. in Different Culture Conditions,” Emerging Microbes & Infections 14, no. 1 (2025): 2508766, 10.1080/22221751.2025.2508766. 40396886 PMC12153010

[49] A. F. Vega‐Benedetti , E. Loi , and P. Zavattari , “DNA Methylation Alterations Caused by Leishmania Infection May Generate a Microenvironment Prone to Tumour Development,” Frontiers in Cellular and Infection Microbiology 12 (2022): 984134, 10.3389/fcimb.2022.984134. 36105147 PMC9465093

[50] A. Schwing , C. Pomares , A. Majoor , L. Boyer , P. Marty , and G. Michel , “Leishmania Infection: Misdiagnosis as Cancer and Tumor‐Promoting Potential,” Acta Tropica 197 (2019): 104855, 10.1016/j.actatropica.2018.12.010. 30529443

[51] A. A. Binhazim , S. S. Shin , W. L. Chapman, Jr. , and J. Olobo , “Comparative Susceptibility of African Green Monkeys (Cercopithecus Aethiops) to Experimental Infection With Leishmania *Leishmania donovani* and Leishmania *Leishmania infantum* ,” Laboratory Animal Science 43, no. 1 (1993): 37–47. 8459677

[52] B. Domínguez‐Asenjo , C. Gutiérrez‐Corbo , Y. Pérez‐Pertejo , S. Iborra , R. Balaña‐Fouce , and R. M. Reguera , “Bioluminescent Imaging Identifies Thymus, as Overlooked Colonized Organ, in a Chronic Model of *Leishmania donovani* Mouse Visceral Leishmaniasis,” ACS Infectious Diseases 7, no. 4 (2021): 871–883, 10.1021/acsinfecdis.0c00864. 33739807

[53] G. G. Jussiani , K. S. Março , P. H. L. Bertolo , R. de Oliveira Vasconcelos , and G. F. Machado , “Thymic Changes due to Leishmaniasis in Dogs: An Immunohistochemical Study,” Veterinary Immunology and Immunopathology 247 (2022): 110416, 10.1016/j.vetimm.2022.110416. 35358749

[54] C. Arrais‐Lima , L. R. Lima , M. G. B. F. Faria , A. Degrossoli , W. W. Arrais‐Silva , and P. C. S. Souto , “Morphological Alterations of Thymus During the Early Murine Leishmaniasis,” Tropical Biomedicine 38, no. 3 (2021): 338–342, 10.47665/tb.38.3.075. 34508341

[55] M. L. Silva‐Freitas , G. Corrêa‐Castro , G. F. Cota , et al., “Impaired Thymic Output Can be Related to the Low Immune Reconstitution and T Cell Repertoire Disturbances in Relapsing Visceral Leishmaniasis Associated HIV/AIDS Patients,” Frontiers in Immunology 11 (2020): 953, 10.3389/fimmu.2020.00953. 32508833 PMC7251171

[56] A. V. A. da Silva , T. L. de Souza , F. B. Figueiredo , et al., “Detection of Amastigotes and Histopathological Alterations in the Thymus of *Leishmania infantum*‐Infected Dogs,” Immunity, Inflammation and Disease 8, no. 2 (2020): 127–139. 32207879 10.1002/iid3.285 PMC7212199

[57] M. Losada‐Barragán , A. Umaña‐Pérez , J. Durães , et al., “Thymic Microenvironment Is Modified by Malnutrition and *Leishmania infantum* Infection,” Frontiers in Cellular and Infection Microbiology 9 (2019): 252, 10.3389/fcimb.2019.00252. 31355153 PMC6639785

[58] M. Losada‐Barragán , A. Umaña‐Pérez , S. Cuervo‐Escobar , et al., “Protein Malnutrition Promotes Dysregulation of Molecules Involved in T Cell Migration in the Thymus of Mice Infected With *Leishmania infantum* ,” Scientific Reports 7 (2017): 45991, 10.1038/srep45991. 28397794 PMC5387407

[59] R. A. Pinna , D. Silva‐Dos‐Santos , D. S. Perce‐da‐Silva , et al., “Malaria‐Cutaneous Leishmaniasis Co‐Infection: Influence on Disease Outcomes and Immune Response,” Frontiers in Microbiology 7, no. 7 (2016): 982, 10.3389/fmicb.2016.00982. 27446022 PMC4921482

[60] N. Goyal , P. Y. Guru , and A. K. Rastogi , “Status of Glutathione in Lymphoid Tissues of Golden Hamster During *Leishmania donovani* Infection,” Indian Journal of Biochemistry & Biophysics 31, no. 3 (1994): 211–213. 7959849

[61] J. P. Rosat , M. Schreyer , T. Ohteki , G. A. Waanders , H. R. MacDonald , and J. A. Louis , “Selective Expansion of Activated V Delta 4+ Cells During Experimental Infection of Mice With *Leishmania major* ,” European Journal of Immunology 24, no. 2 (1994): 496–499, 10.1002/eji.1830240237. 8299701

[62] C. E. Corbett , R. A. Paes , M. D. Laurenti , H. F. Andrade Júnior , and M. I. Duarte , “Histopathology of Lymphoid Organs in Experimental Leishmaniasis,” International Journal of Experimental Pathology 73, no. 4 (1992): 417–433. 1390190 PMC2002360

[63] I. A. Eltoum , E. E. Zijlstra , M. S. Ali , et al., “Congenital Kala‐Azar and Leishmaniasis in the Placenta,” American Journal of Tropical Medicine and Hygiene 46, no. 1 (1992): 57–62, 10.4269/ajtmh.1992.46.57. 1536385

[64] L. Schnur , A. Zuckerman , and B. Montilio , “Dissemination of Leishmanias to the Organs of Syrian Hamsters Following Intrasplenic Inoculation of Promastigotes,” Experimental Parasitology 34, no. 3 (1973): 432–447, 10.1016/0014-4894(73)90103-3. 4773580

[65] V. Middelkamp and E. Kekäläinen , “Measuring Thymic Output Across the Human Lifespan: A Critical Challenge in Laboratory Medicine,” Geroscience 47, no. 6 (2025): 6797–6806, 10.1007/s11357-025-01555-3. 39946072 PMC12638580

[66] M. S. Ventevogel and G. D. Sempowski , “Thymic Rejuvenation and Aging,” Current Opinion in Immunology 25, no. 4 (2013): 516–522, 10.1016/j.coi.2013.06.002. 23831111 PMC3775968

[67] T. Wertheimer , E. Velardi , J. Tsai , et al., “Production of BMP4 by Endothelial Cells Is Crucial for Endogenous Thymic Regeneration,” Science Immunology 3, no. 19 (2018): eaal2736, 10.1126/sciimmunol.aal2736. 29330161 PMC5795617

[68] E. Velardi , J. J. Tsai , A. M. Holland , et al., “Sex Steroid Blockade Enhances Thymopoiesis by Modulating Notch Signaling,” Journal of Experimental Medicine 211, no. 12 (2014): 2341–2349, 10.1084/jem.20131289. 25332287 PMC4235646

[69] D. Granadier , L. Iovino , S. Kinsella , and J. A. Dudakov , “Dynamics of Thymus Function and T Cell Receptor Repertoire Breadth in Health and Disease,” Seminars in Immunopathology 43, no. 1 (2021): 119–134. 33608819 10.1007/s00281-021-00840-5 PMC7894242

[70] J. A. Dudakov and M. R. M. van den Brink , “Burning Down the House: Thymic Repair and Regeneration After Acute Damage,” Immunological Reviews 332, no. 1 (2025): e70050. 40579877 10.1111/imr.70050 PMC12376859

[71] A. Morales‐Sánchez , M. Lavaert , M. S. Vacchio , et al., “Enhancing Thymic Function Improves T‐Cell Reconstitution and Immune Responses in Aged Mice,” PLoS Biology 23, no. 7 (2025): e3003283, 10.1371/journal.pbio.3003283. 40720380 PMC12303306

